# Analysis of the n-GaN electrochemical etching process and its mechanism in oxalic acid

**DOI:** 10.1039/d1ra07992a

**Published:** 2022-02-07

**Authors:** Artem Shushanian, Daisuke Iida, Zhe Zhuang, Yu Han, Kazuhiro Ohkawa

**Affiliations:** Physical Science and Engineering (PSE) Division, King Abdullah University of Science and Technology (KAUST) Thuwal 23955-6900 Kingdom of Saudi Arabia; Computer, Electrical and Mathematical Sciences and Engineering (CEMSE) Division, King Abdullah University of Science and Technology (KAUST) Thuwal 23955-6900 Kingdom of Saudi Arabia kazuhiro.ohkawa@kaust.edu.sa

## Abstract

We studied the wet electrochemical etching of n-GaN films in oxalic acid. The electrooxidation processes occur in a potentiostatic mode in the voltage range of 5 to 20 V. We described the formation of the porous n-GaN layer structures in several ways. Firstly, we observed the microphotographs of the cross section to characterize the nanostructure. Secondly, we examined the reaction products in a liquid phase using ICP-OES and TOC-TN methods, while vapor-phase products were examined by gas chromatography. Finally, according to the product data analysis, we demonstrate a mechanism for the electrochemical oxidation of n-GaN in oxalic acid, which involves 6 electrons.

## Abstract 2

We studied the mechanism of wet electrochemical etching of n-GaN films in oxalic acid.

## Graphical abstract

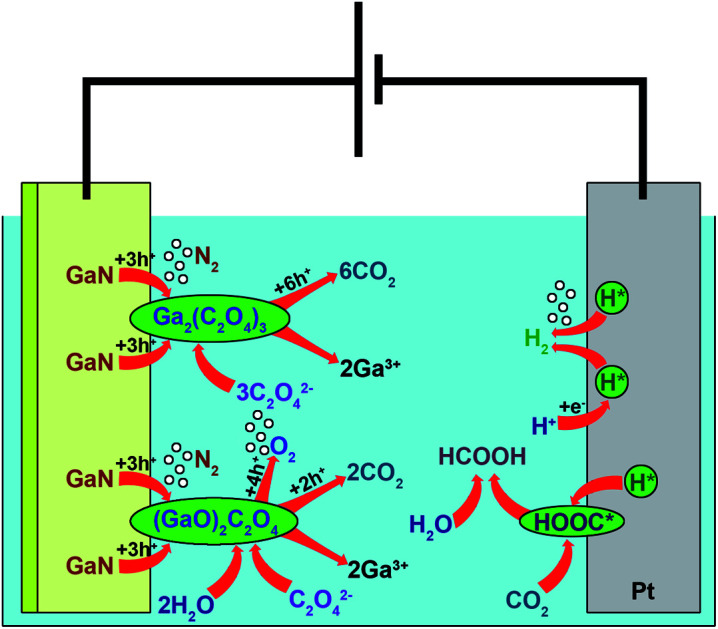

## Introduction

Generally, III-nitride systems are notable for their high chemical and thermal stability,^1–5^ wide tunable bandgap (0.67–6.04 eV)^6–8^ and the possibility of their nanoscale processing^9–12^ that make them a promising basis for solid-state lighting devices. Recent fabrication technology is a useful option to modify the structure of III-nitride systems to selectively etch excessively n-doped layers either for their complete destruction and lift-off of the remaining structure^13,14^ or for their porosification.^15–18^

The formation of porous layers may help to improve the properties of III-nitride-based devices in various ways. For example, it can relax the lattice strain in fine heterogeneous film structures and lead to enhanced emission at longer wavelengths.^19,20^ Furthermore, the design of periodic n^+^-doped and unintentionally (uid)-doped III-nitride layers provides an opportunity to create mirrors based on an air-gap distributed Bragg reflector (DBR), which may be useful for increasing the light extraction efficiency of light-emitting diodes (LEDs).^21^ Encapsulation of various gases and liquids in porous structures is also promising for the attainment of reversible changes of properties in such systems.^22^

There are plenty of methods for manufacturing porous III-nitride materials. Dry etching methods are widely used and include reactive ion etching^23,24^ and hydrogen etching.^25^ However, dry etching treatment may cause a few unintended damages to samples. Electroless wet etching is also a promising approach, but it often requires processing and post-processing of the samples using toxic substances at high concentrations, such as hydrofluoric and nitric acids.^26,27^

Photoelectrochemical (PEC) wet etching is one of the most studied methods of III-nitride porosification due to its precise process control and eco-friendly equipment. However, problems may appear in larger scale production of porous III-nitrides, such as the need for uniform ultraviolet illumination of bigger samples. PEC etching is described as a surface reaction due to photoabsorption,^28^ whereas an electrochemical (EC) oxidation process is supposed to occur all over the volume of n^+^-doped layers.

Despite the uniformity and simplicity of the EC etching process, the chemical mechanisms that have been proposed are based on either assumptions and predictions^29,30^ or on experiments that involve Xe-lamp irradiation of the sample.^28,31,32^ However, with a lack of product analysis, these studies agree that GaN etching has a 3-electron nature:

1 2GaN → 2Ga^3+^ + N_2_ + 6e^−^

Clear understanding this mechanism is a basic step in the design of porous III-nitride-based devices. Therefore, to describe the III-nitride electrooxidation process, we need to choose the right conditions to investigate it reliably in the future. Thus, we consider the etching of n-GaN layers in a potentiostatic mode presented in a two-electrode system.

In this paper, we present the detailed mechanism of the EC oxidation reaction of n-GaN in oxalic acid based on liquid- and vapor-phase physicochemical analyses. The transformation of the oxalic anion indicates a chemical interaction of the GaN surface with the solvent and the formation of intermediate products. Moreover, we demonstrate the contribution of a Pt cathode to the reaction mechanism. The experimental configuration used in this study is common among research groups,^22,33–36^ and hence, the results could address the requirements for the realization of device production with porous III-nitride architecture elements.

## Experimental

The sample structure considered is a uid-GaN cap layer (100 nm, *n* ∼ mid. 10^16^ cm^−3^) deposited on n-GaN : Si (3.0 μm, *n* = 3 × 10^18^ cm^−3^) on uid-GaN (2.3 μm), which was grown on a *c*-plane patterned sapphire substrate (PSS) using organometallic vapor phase epitaxy. For the EC oxidation of GaN, we set up a 2-electrode system with a Pt wire as the counter electrode, which was located 2 cm away from the sample. The etching occurred in 0.25 M oxalic acid (pH ≈ 1) at room temperature and lasted for 30 min. We dissolved oxalic acid dihydrate (VWR Chemicals, 99.99%) in deionized water (MilliQ) to prepare the electrolyte solution, the volume of which was 50 mL in every experiment.

**Fig. 1:**
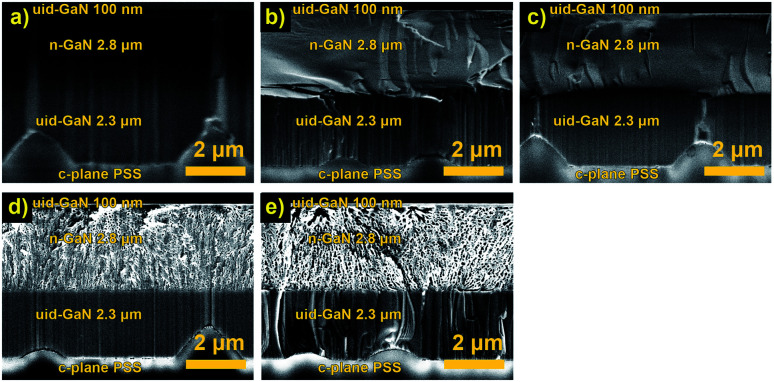
Cross-sectional SEM images of (a) unetched n-GaN and etched n-GaN after 30 min of etching in 0.25 M oxalic acid at (b) 5 V, (c) 10 V, (d) 15 V, and (e) 20 V.

The loss of GaN was calculated according to the concentration of Ga in the electrolyte solution every 5 min. The amount of elemental Ga was estimated using measurements by inductively coupled plasma-optical emission spectrometry (ICP-OES), which were performed using an Agilent Varian 7200-ES. The calibration curve was obtained with 10% uncertainty at wavelengths corresponding to Ga emission: 250.019 nm, 287.423 nm, 294.363 nm, and 417.204 nm. We also considered that the lab equipment contributed 3% more uncertainty during sample-volume measurements, and hence, the total uncertainty for ICP-OES method is 13%.

We observed the sample microstructures before and after the etching process by cross-sectional SEM (FEI Magellan). The gas generated at the cathode and anode parts was analyzed using a gas chromatograph (Shimadzu GC-8A) with thermal conductivity detection. The nitrate and inorganic carbon contents in the electrolyte after the experiment were defined by measurements by the inorganic carbon (IC) and total nitrogen (TN) analyzers (Shimadzu TOC-L CSH). The IC was measured according to two calibration curves at 0–50 ppm and 0–10 ppm, and for TN, we obtained a calibration curve at 0–5 ppm. We connected the electrodes to a power source (Keysight B2901A) to apply bias to the system and record the current changes. Cyclic voltammetry (CV) measurements were performed in a 3-electrode system with a standard silver chloride electrode (SSCE) as the reference one. We recorded the CVs of the n-GaN working electrode in 0.05 M sulfuric acid (Fisher Scientific, 95.0–98.0 w/w%), 0.05 M sulfuric acid solution in the presence of 2.5 mM oxalic acid and in 0.25 M oxalic acid solution.

## Results

Before the experiment, the border between uid-GaN and n-GaN layers could be slightly distinguished, as shown in Fig. 1a. After the experiments, all the samples showed a uniform n-GaN layer, and no etching of the cap layer occurred (Fig. 1c–e). This suggests that a uniform EC reaction was obtained in the volume during the whole exposure time.

At 5 V and 10 V, the etching proceeded slowly, and we observed no formation of porous structures for 30 min. At 15 V and 20 V, the sizes of pores increased accordingly, while the pore structure and shape remained similar (Table 1). The pores appeared to be branching and current-oriented, as expected at relatively high voltages and carrier concentrations.^17^ Furthermore, the reaction proceeds uniformly throughout the whole layer thickness.

**Table 1:** Pore structure characterization^a^

Voltage (*V*)	Pore density in cross section (cm^−2^)	Pore size (nm)	Average pore size (nm)	Porosity by SEM (%)
15	1.3 × 10^10^	10–90	30	11.7
20	7.6 × 10^9^	10–300	57	32.9

a
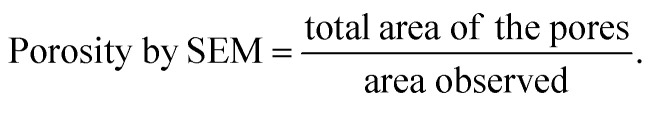

**Fig. 2:**
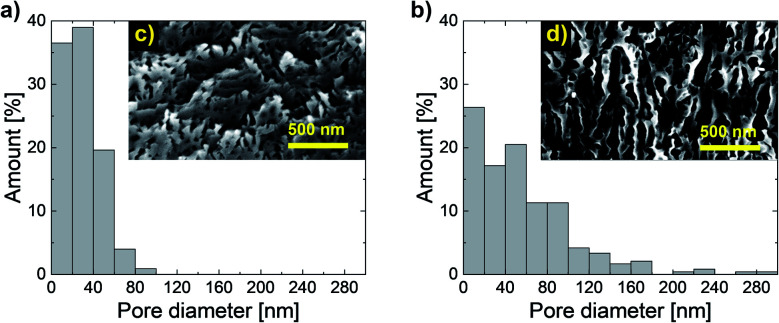
Pore size distribution after EC etching at (a) 15 V and (b) 20 V and the corresponding SEM images of cross-sectional areas for pore size and distribution calculations after the (c) 15 V and (d) 20 V experiments.

**Fig. 3:**
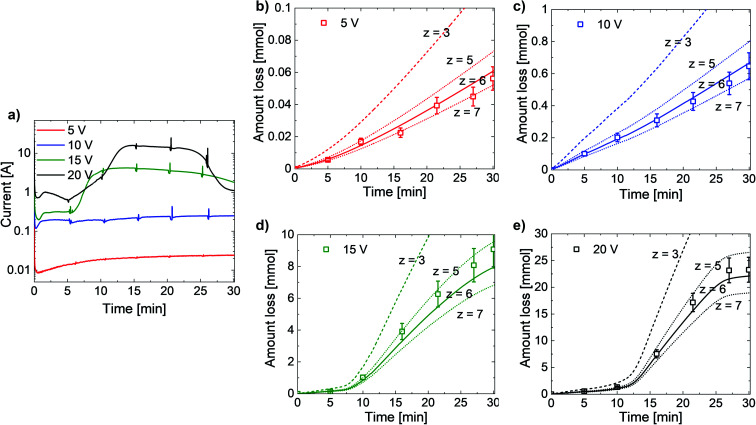
(a) Etching current changes, (b–e) GaN amount losses (squares) measured by ICP-OES, and GaN amount losses estimated from current at *z* = 3 (dashed), *z* = 6 (solid), *z* = 5, and *z* = 7 (upper and lower dotted lines, respectively) at (b) 5 V, (c) 10 V, (d) 15 V, and (e) 20 V. All values are normalized to 1 cm^3^ of GaN.

We measured the porosities of our samples in two ways. The first one was based on the ratio of the total area of pores and the total area observed (Table 1 and Fig. 2). The second one involved the estimation of the ratio of the GaN amount that is transferred to the liquid phase and the total amount of the GaN layer before the experiment (Table 2 and Fig. 3).

**Table 2:** Charge, amount of transferred electrons calculated, and porosity characterization

Voltage (*V*)	GaN loss (*n*)^a^ (mmol)	Charge (*Q*)^a^ (C)	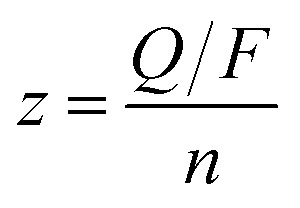	Porosity by GaN loss (%)
5	0.056 ± 0.007	35.59	6.5 ± 0.8	0.07 ± 0.01
10	0.66 ± 0.08	393.96	6.3 ± 0.8	0.8 ± 0.1
15	9.1 ± 1.2	4635.8	5.3 ± 0.7	11.5 ± 1.5
20	23.3 ± 3.0	12 812	5.7 ± 0.7	29.6 ± 3.8

a The values are normalized to 1 cm^3^ of GaN.

According to the SEM observations, the porosities of n-GaN were 11.7% and 32.9% after etching at 15 and 20 V, respectively. Fig. 2 shows the pore size distribution. More than 50% of the pores generated were less than 60 nm in diameter for both samples, but there was a higher variety in the pore size with the increase in voltage.

As for the porosity estimations, we again used two approaches to describe the EC etching reaction. The integration time of the reaction current gives the total charge (eqn (2)), while the amount of GaN that took part in the EC process can be found from the ICP-OES measurements. Hence, we can connect these data *via* the Faraday constant and extract the amount of electrons (*z*) needed to transfer one gallium atom from a single crystal to the electrolyte solution (eqn (3)):2
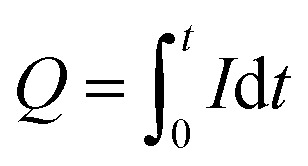
3
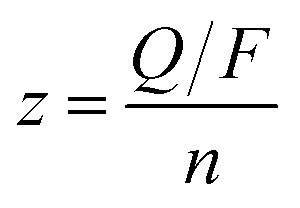
where *Q* (C) is the total charge of the etching reaction, *I* (A) is the current, *t* (s) is the reaction exposition time, *z* is the number of electron–hole pairs responsible for the transition of one Ga atom from a single crystal to the electrolyte, *F* is the Faraday constant (96 485 C mol^−1^), and *n* (mol) is the amount loss of GaN calculated according to the ICP-OES results (Table 2). We calculated the n-GaN layer porosities for all experiments as the ratio of the GaN amount losses to the amounts of all n-GaN layers. The values estimated using SEM and the GaN loss matched closely.

Fig. 3 presents the changes in etching current and n-GaN decomposition to a liquid phase. Sharp peaks on current plots (Fig. 3a) are caused by the extraction of some electrolytes for further ICP-OES measurements of Ga concentrations. The etching current and GaN loss increased by approximately one order of magnitude with every 5 V increase in voltage except at 20 V, at which the sample showed exhaustion of the reaction after ∼23 min. At 5 V and 10 V, we observed the constant linearity of the EC reaction throughout the exposure time. However, at higher voltages, the reaction rate changed after ∼7 min at 15 V and after ∼13 min at 20 V. Thus, at 15 V and 20 V, the etching reaction is diffusion-controlled at the beginning, and changes to activation-controlled as the generated pores start to provide enough solvent all over the GaN layer for a higher reaction rate. We compared the data obtained from current measurements and the amounts of elemental gallium found in solutions after the experiments, which indicated correspondence to a 6-electronic reaction within the uncertainty of 13%. The results of our measurements alter from the former idea of 3-electronic electrooxidation of GaN (eqn (1)). Furthermore, the reaction is considered to proceed the same way at any rate, and hence, the values of *z* = 5 and *z* = 7 do not represent the behavior of electrooxidation of GaN within the whole voltage range of 5–20 V.

**Fig. 4:**
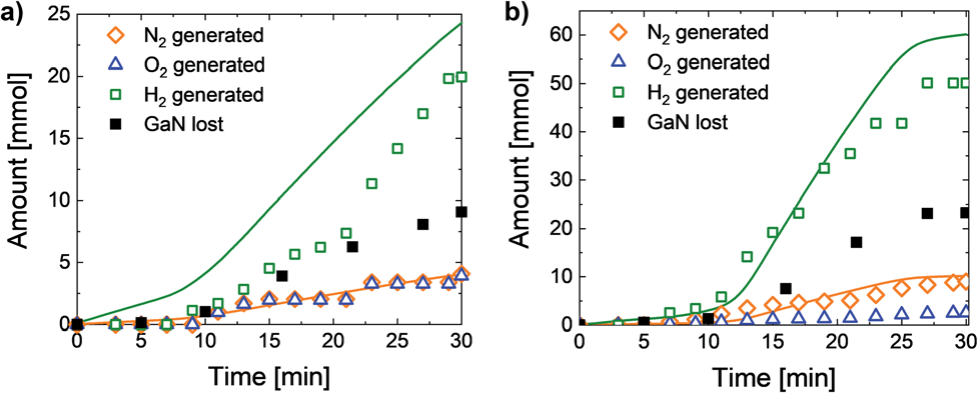
N_2_ and O_2_ gas generation on the anode, H_2_ generation on the cathode and GaN loss measured by ICP-OES at (a) 15 V and (b) 20 V. N_2_ expected on the anode and H_2_ expected on the cathode at *z* = 6 (solid lines). All values are normalized to 1 cm^3^ of GaN.

The gas generation during the 15 V and 20 V reactions is plotted in Fig. 4. The amount of gas generated at 5 V and 10 V was unobservable. Chromatographic analysis of the vapor phase (Table 3) shows that nitrogen and oxygen are the main components of the gas generated at the anodic part of the reaction, and hydrogen is the main one at the cathodic part. The hydrogen impurities that form at the anode may be a result of the self-oxidation of water. The nitrogen generated at the cathode is probably a product of a side reaction of nitrate forms that undergo reduction.

**Table 3:** Gas phase composition

Voltage (*V*)	Electrode	H_2_ (%)	O_2_ (%)	N_2_ (%)
15	Anode	0.2	48.9	50.9
	Cathode	92.3	0	7.7
20	Anode	2.1	29.3	68.6
	Cathode	93.4	0	6.6

We observed the change in the diffusion control to the activation control of the reaction as the gas generation plots bend at ∼7 min for the 15 V reaction and at ∼13 min for the 20 V reaction. It corresponds with our analysis of the reaction rate according to the Ga concentration in the electrolyte.

After both 15 V and 20 V experiments, the final amount of nitrogen was nearly twice as low as the equimolar amount of elemental Ga found in the electrolyte by ICP-OES measurements: *n*_GaN_ : *n*_N_2__ = 2 : 0.9 at 15 V (Fig. 4a) and *n*_GaN_ : *n*_N_2__ = 2 : 0.8 at 20 V (Fig. 4b), as we expected while GaN was the only nitrogen source in the system. At *z* = 6, we assumed that the reduction of 6 protons on the cathode would lead to the generation of the amount of hydrogen that is 6 times more than the amount of nitrogen. However, the final amount of hydrogen was close to 5 times more than the amount of nitrogen: *n*_H_2_ _: *n*_N_2__ = 5 : 1 at 15 V (Fig. 4a) and *n*_H_2_ _: *n*_N_2__ = 5 : 0.9 at 20 V (Fig. 4b). The reason for this phenomenon is described in the discussion of the reaction mechanism. Etching at 15 V produced the same amount of oxygen and nitrogen, whereas at 20 V, the amount of O_2_ was significantly less. This indicates a decrease in the water-oxidation part of the reaction and the appearance of an alternative process that becomes dominant at a higher voltage.

IC/TN analysis of the electrolyte (Table 4) was carried out immediately after the experiments and also after one week of storage in an oxygen atmosphere. According to Rumpf *et al.*,^37^ all carbon dioxide that may form during the reaction is expected to be found dissolved in the liquid phase. A blank sample with the electrolyte was used as a background solution to prevent the overestimation of aqueous CO_2_ (CO_2aq_) that could come from the oxidation of oxalic acid, which happens much more slowly than the oxidation of formic acid that we expect to observe.^38^ The concentration of CO_2aq_ in a blank electrolyte before and after storage in oxygen did not change and was equal to 0.30 ppm.

**Table 4:** Inorganic carbon and total nitrogen amounts in the liquid phase^a^

Voltage (*V*)	IC (mmol)		TN (mmol)	
	As-etched	One week after	As-etched	One week after
5	0.016 ± 0.002	0.33 ± 0.04	0.0056 ± 0.0007	0.0070 ± 0.0009
10	0.069 ± 0.009	1.41 ± 0.18	0.056 ± 0.007	0.049 ± 0.006
15	0.10 ± 0.01	1.63 ± 0.19	0.17 ± 0.02	0.17 ± 0.02
20	0.12 ± 0.01	1.37 ± 0.18	0.37 ± 0.05	0.44 ± 0.05

a All values are normalized to 1 cm^3^ of GaN.

The amounts of inorganic carbon in fresh electrolytes were insignificant, which means that the assumption of CO_2aq_ formation is not obvious. However, we observed an increase in the inorganic carbon concentration in the solution after one week. We consider this CO_2aq_ to be a result of the surface interaction of GaN and the electrolyte. The concentration of nitrate forms remains the same after storage in oxygen for one week.

We recorded the CVs in three different solutions: 0.05 M sulfuric acid (open circuit potential (*E*_oc_) was −0.42 V *vs.* SCCE), 0.05 M sulfuric acid + 2.5 mM oxalic acid (*E*_oc_ = −0.39 V *vs.* SCCE) and 0.25 M oxalic acid (*E*_oc_ = −0.34 V *vs.* SCCE) (not shown here). However, the concentration of oxalic acid is too high to run qualitative measurements under conditions similar to experimental ones. Nevertheless, according to the data recorded during the CV analysis, we may qualify the nitrogen formation that appears in sulfuric acid as well as in the presence of oxalic acid. We registered an additional peak of oxalate anion oxidation on the voltammogram recorded in the presence of oxalic acid, as this peak does not appear during the measurements in a pure sulfuric acid solution.

## Discussion

The measured loss of GaN showed a close correspondence with the theoretical result at *z* = 6. Moreover, for GaP and GaAs, EC decomposition is supposed to involve 6 electrons.^39^ The similarity of the GaN, GaP, and GaAs etching processes suggests the formation of NO_2_^−^ anions rather than N_2_ generation as the prevailing reaction product. Nevertheless, the amounts of nitrate forms (Table 4) show that this process is a side reaction that takes 2–13% of the total reaction charges, and its impact is significantly less at higher voltages.

We consider all the oxygen adsorbed on the anode surface to take part in the reaction, as no oxygen is expected to remain on it afterwards.^40^ This is why one of the reaction paths involves the formation of Ga–O bonds on the reaction surface. Since 3 electrons are lost by elemental nitride while dinitrogen is being generated (eqn (4)) and the elemental oxide fragment needs to lose 2 electrons for further dioxygen generation, one remaining electron is involved in the interaction with the oxalate anion (eqn (5)). Therefore, the 15 V reaction occurs with intermediate oxide formation almost completely (eqn (4) and (5)), as the amounts of nitrogen and oxygen generated at 15 V are equimolar (Table 3 and Fig. 4a). At 20 V, the fraction of oxygen is 29.3% of total gas mixture (Table 3 and Fig. 4b), and hence, nitrogen generated the way described in eqn (4) and (5) is 42.7% of its total final amount. The alternative path of 20 V reaction proceeds simultaneously with (4) and (5), but no oxygen is involved (eqn (6)). Therefore, after 3 electrons spent on nitride oxidation, the remaining 3 electrons participate in the interaction with the oxalic anion (eqn (7)). Thus, the anodic part of the EC reaction occurs as follows:

4 2GaN + 2H_2_O + C_2_O_4_^2−^ → [(GaO)_2_C_2_O_4_]_ads_ + N_2_↑ + 4H^+^ + 6e^−^

5 [(GaO)_2_C_2_O_4_]_ads_ → 2Ga^3+^ + O_2_↑ + 2CO_2aq_ + 6e^−^

An alternative way with no oxygen participating in the reaction also involves 6 electrons:

6 2GaN + 3C_2_O_4_^2−^ → [Ga_2_(C_2_O_4_)_3_]_ads_ + N_2_↑ + 6e^−^

7 [Ga_2_(C_2_O_4_)_3_]_ads_ → 2Ga^3+^ + 6CO_2aq_ + 6e^−^

The side reaction occurs in the same way as for GaP and GaAs:^39^

8 GaN + 2H_2_O → Ga^3+^ + NO_2_^−^ + 4H^+^ + 6e^−^

Considering the process that occurs on the cathode, we have to note two facts. The first one is that the amount of hydrogen generated is lower than expected at *z* = 6, as mentioned above. Second, the electrolyte does not contain a significant amount of carbon dioxide after the etching experiments in the whole voltage range, but it is found after one week of exposure to oxygen. We also took into account the fact that dissolved carbon dioxide has a strong tendency toward chemisorption on a Pt cathode, and the Gibbs energy of CO_2aq_ adsorption is a quadratic function of the potential in the system.^41^ Although no direct reduction of CO_2_ occurs on the cathode, the interaction with hydrogen leads to the formation of a carboxylic radical.^42^

Vassiliev *et al.*^43^ describe this process as a limiting reaction in a series of interactions of adsorbed carboxylic radical ([COOH*]_ads_) with hydrogen dissolved in Pt. In our case, a high reaction rate and the limited amount of cites to adsorb CO_2_ on the electrode further interrupt the [COOH*]_ads_ reduction by adsorbed hydrogen ([H*]_ads_). Thus, the mechanism of the GaN etching reaction on the Pt cathode is as follows:

9 6H^+^ + 6e^−^ → 6[H*]_ads_^43^

10 CO_2aq_ + [H*]_ads_ → [COOH*]_ads_^43^

11 5[H*]_ads_ → 2.5H_2_↑

Further reaction of carboxylic radicals with the environment probably tends to lead to the formation of formic acid as follows:

12 [COOH*]_ads_ + H_2_O → HCOOH + OH*

Formic acid is known as a strong reductant, and it slowly reacts with oxygen as follows:^44^

13 HCOOH + 0.5O_2_ → H_2_O + CO_2aq_^44^

The IC measurements made at different times after the experiment qualitatively indicate the scheme of CO_2_ transformations before being registered.

According to the nitrates found after the reaction in the liquid phase and the nitrogen gas that appeared on cathode, the side reaction is as follows:

14 NO_2_^−^ + H^+^ → [HNO_2_]_ads_

15 2[HNO_2_]_ads_ + 6H^+^ + 6e^−^ → N_2_↑ + 4H_2_O

**Fig. 5:**
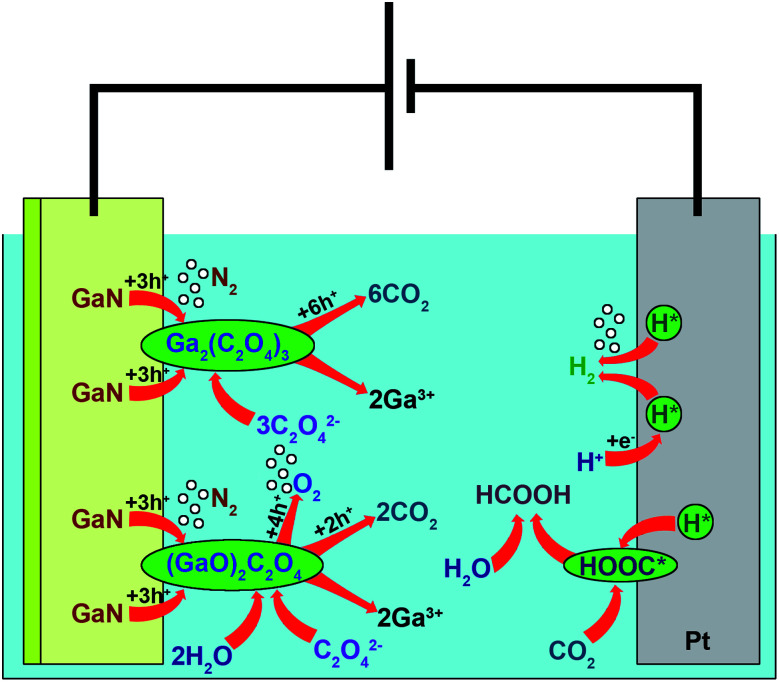
A schematic representation of n-GaN etching in oxalic acid.

Thus, the mechanism of electrooxidation of GaN (Fig. 5) differs from the ones studied before (eqn (1)),^28–32^ and it is believed that it involves 6 electrons, as proposed for GaP and GaAs.^39^ However, we present a notable remark: unlike the formation of XO_2_^−^ (X = N, P, and As), an alternative process occurs. After the oxidation of nitride to nitrogen gas, we assume that the oxidation of solvent components occurs — namely, oxalate anions and water molecules in our case, which are absorbed in the n-GaN layer. The generation of hydrogen gas is accompanied by the interaction of adsorbed hydrogen and oxidized forms of the solvent components.

## Conclusions

We have performed a series of EC etching experiments of n-GaN films at different biases and observed the compositions of the vapor and liquid phases. Electrooxidation of the n-GaN layer leads to its transformation into a uniform porous structure with branching current-oriented pores. The results of a number of quantitative physicochemical analysis methods of the products indicated a 6-electron nature of the etching reaction and fully revealed the mechanism of EC oxidation of n-GaN, which includes the formation of intermediates formed by adsorption of the solvent on the GaN surface. Thus, the reaction mechanism is uniform throughout the whole volume and is valid in the observed voltage range. An in-depth understanding of this reaction lays a solid foundation for the investigation of transformations of more complicated III-nitride systems for low-damage and high-precision processing of nitride-based devices.

## Author contributions

Artem Shushanian: investigation, methodology, writing – original draft; Daisuke Iida: validation, writing – review & editing; Zhe Zhuang: validation, writing – review & editing; Yu Han: supervision, writing – review & editing; Kazuhiro Ohkawa: conceptualization, project administration, writing – review & editing.

## Conflicts of interest

There are no conflicts to declare.

## Supplementary Material
